# The Potential Role of Psilocybin in Traumatic Brain Injury Recovery: A Narrative Review

**DOI:** 10.3390/brainsci15060572

**Published:** 2025-05-26

**Authors:** Charles Palmer, Ally T. Ferber, Brian D. Greenwald

**Affiliations:** 1 Hackensack Meridian School of Medicine, Nutley, NJ 07110, USA; charles.palmer@hmhn.org; 2 Department of Physical Medicine and Rehabilitation, JFK Johnson Rehabilitation Institute at Hackensack Meridian Health, Edison, NJ 08820, USA; ally.ferber@hmhn.org

**Keywords:** medical psilocybin, traumatic brain injury, addiction, psychedelic

## Abstract

**Background:** This narrative review explores psilocybin’s potential use as a therapeutic agent in patients with traumatic brain injury (TBI). **Methods:** We engaged in a search of PubMed, ScienceDirect, and Cochrane’s databases for information on the effects of psilocybin. We also reviewed articles where psilocybin was used in patients with TBI. Articles from 2000–2025 were included. **Results:** A total of 29 articles met our initial inclusion criteria. Additionally, 13 articles were obtained from reference lists and 3 more articles on the legality of psilocybin from public websites. **Conclusions:** Assisted psilocybin use may have benefits in TBI by reducing inflammation, promoting neuroplasticity and neuroregeneration, and alleviating associated mood disorders. Positive findings in related fields, like treatment for depression and addiction, highlight the necessity for more extensive clinical trials on psilocybin’s role in TBI recovery.

## 1. Introduction

Approximately 69 million people worldwide suffer from traumatic brain injuries (TBIs) each year. The incidence was highest in North America, with rates reaching 1299 out of 100,000 people [1]. Progress in modern medicine has increased the survival rate of TBI patients [2]. However, there continues to be a need for new treatment solutions for TBI survivors because of their rising numbers, since these survivors experience extended physical, cognitive, and psychological difficulties.

TBI is the result of direct physical impact that leads to neuronal necrosis and tissue damage. The initial impact creates a primary injury, otherwise known as a contusion. The contusion can later expand, resulting in delayed neurodegeneration [3]. The area around the contusion, known as the pericontusion, can also expand due to edema or increasing pressure in the brain, increasing the risk of ischemia [3]. The impact is also followed by an immune response involving macrophages. Macrophages help clear cellular debris and support tissue repair, but their prolonged release of pro-inflammatory mediators can worsen neuronal death, increase neurovascular injury, and lead to long-term white matter loss [4]. Macrophages can polarize into either pro-inflammatory (M1) or anti-inflammatory (M2) states. Early after TBI, both M1 and M2 markers are present, but the expression of M2 quickly declines [4]. The M1 state is longer lasting, leading to the unopposed release of pro-inflammatory cytokines and resulting in neurodegeneration. A method that can address the neurodegeneration and inflammation found in TBI would be a crucial step forward in finding effective treatments for TBI patients.

A recent trend is the question of psychedelics’ role in the clinical setting. Because of their structural similarity to tryptophan, psychedelics can interact with serotonin receptors, functioning as agonists on the 5HT-2A receptor [5]. Psychedelics have shown promise in treating substance use disorders (SUDs) and mood disorders through interaction with the dopamine and serotonin pathways as well as the hypothalamic–pituitary–adrenal (HPA) axis [5]. This new trend, along with the increasing need for TBI treatments, prompted the question of if psychedelics have a potential role in TBI treatment, given the similar neurochemical disturbances found in patients after a traumatic brain injury. Previous explorations into this question have involved consideration of multiple psychedelics such as LSD, MDMA, ketamine, and psilocybin with positive indications yielded [6,7]. This paper aims to focus on psilocybin alone, creating a focused review. Psilocybin is a natural psychedelic alkaloid found in over 100 species of mushrooms in the Psilocybe genus [5]. When it comes to treatment with psilocybin, there is an initial concern of the legality. In the United States, nine states have already decriminalized it, and two states have legalized assisted medical use. During this time, there have been trials on its use for depression, anxiety, and substance use disorder, but there are no data as of yet on its direct application in TBI patients. This paper explores the therapeutic potential of psilocybin specifically, looking at examples of its anti-inflammatory and synaptogenesis-inducing properties and how these benefits could play a role in TBI treatment.

## 2. Methods

**Figure 1:**
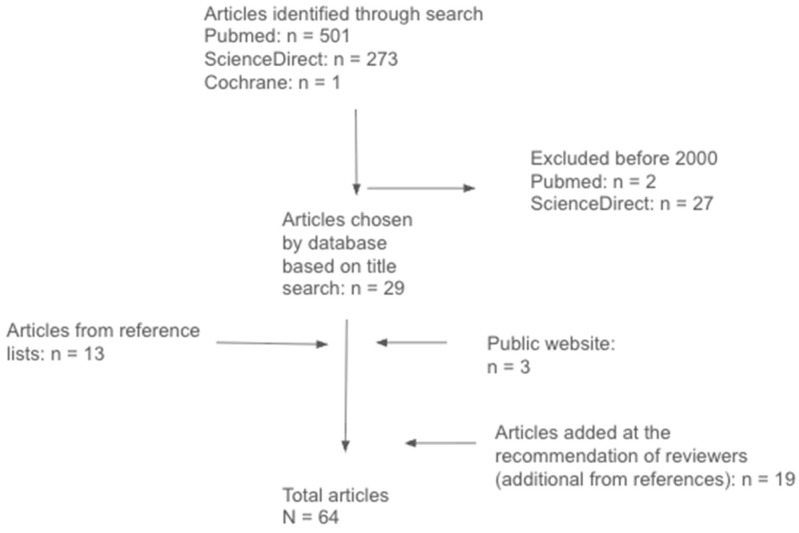
The depicted method behind the inclusion and exclusion of the studies used in this review.

This narrative review carried out its search for articles on online databases, including PubMed, ScienceDirect, and Cochrane. Publications considered were from 2000 to 2025 and were found with the following search phrases: psilocybin, traumatic brain injury (TBI), indications, mechanism of action, neuroplasticity, and inflammation. Combinations of these terms were used as well: “psilocybin and TBI”, “psilocybin mechanism of action”, “psilocybin neuroplasticity”, and “psilocybin inflammation”. The search lasted from August 2024 to January 2025. Articles included were systematic reviews, meta-analyses, randomized controlled trials, and cohort studies that explained psilocybin’s biochemistry, pharmacodynamics, and therapeutic potential. Articles were excluded due to repetitive information from previously included articles. Additional references were gathered from the bibliographies of relevant articles and at the recommendation of reviewers. Public websites were also included and contained information on the legal status of psilocybin in the United States [Figure 1]. Only publications in English or their translated versions were included.

## 3. Legality

When considering the use of psilocybin in a medical setting, it is important to consider the current legal status and how this has evolved over time. In 2019, Denver, Colorado set itself as the first city in the United States to decriminalize psilocybin. This began a trend, which spread to cities like Oakland, Santa Cruz, Ann Arbor, and Washington, D.C. [8]. Shortly after, in 2020, Oregon became the pioneer state to legalize psilocybin for medically supervised use, followed two years later by Colorado [8,9]. This movement encouraged other states, Massachusetts among them, to start planning their own legalization initiatives in 2024.

**Figure 2:**
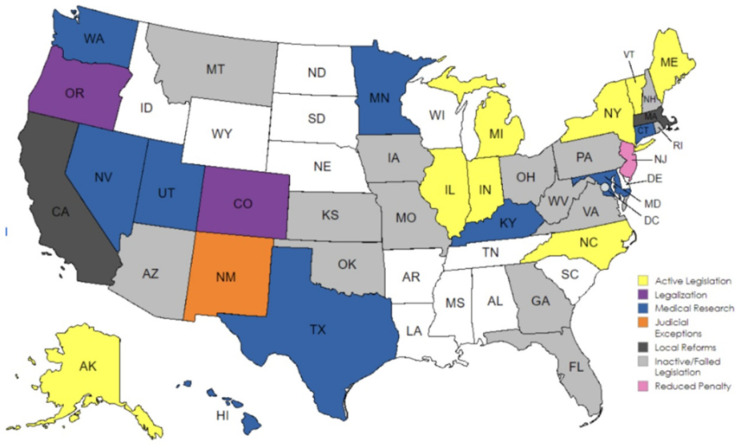
Map with the states and their current status in regard to psilocybin legalization [10].

**Figure 3:**
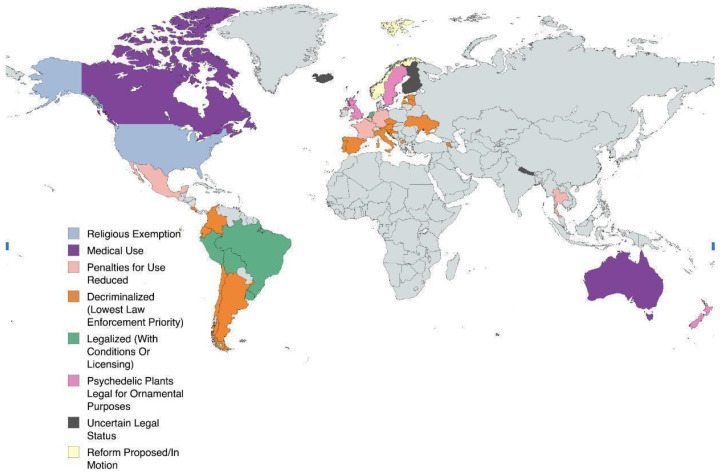
Map showing the current psilocybin legalization status of countries across the world [11].

Currently, psilocybin is decriminalized in nine states and allowed for medical use in two. Using the progression of cannabis legalization, Siegal et al. built an analytic model to predict the course of psychedelic reform. The model predicts a majority of states will legalize psychedelics by 2034 or 2037 [9]. The current status of legalization in the United States and internationally is seen in Figure 2 and Figure 3 [10,11].

## 4. Indications

There are currently no FDA indications for psilocybin treatment. Most research on the clinical applications of psychedelics investigate their ability to treat mood and anxiety disorders [12]. Psilocybin has already proven to be effective in treating mood disorders following a cancer diagnosis [13]. Another study found psilocybin to reduce depressive symptoms in a population of individuals resistant to standard therapies [14,15].

As a controlled substance and hallucinogenic, there are concerns that using psilocybin in the medical setting could lead to substance use disorder. However, psilocybin has shown promise as a treatment for substance use disorder. Combining psilocybin with Motivation Enhancement Therapy significantly increased abstinence rates (*p* < 0.05) in those with alcohol use disorder with no significant adverse events reported [16]. Another trial evaluated psilocybin’s use as a smoking cessation aid. At their 12-month follow-up, 67% of participants were confirmed as smoking abstinent. At long-term follow-up (mean of 30 months), 60% maintained abstinence [17]. The available research is indicative of positive outcomes in psilocybin-assisted addiction therapy, but concerns about addiction should not be overlooked by clinicians, and there is still a need for caution in its clinical use.

In most clinical trials, psilocybin is administered through oral routes either as psilocybin isolate, synthetic psilocybin, or dried *Psilocybe cubensis* [18]. The initial trials administered psilocybin based on the weight of the patient. However, weight-based doses did not produce significant differences [18]; thus, current trials use fixed doses [19].

Based on a review by Johnson et al. [12], therapeutic psilocybin should be administered in a controlled environment to ensure safety and efficacy. There are three main components of psilocybin therapy outlined by Johnson: careful participant screening, preparatory sessions, and close monitoring during sessions [12]. The screening process excludes those at increased risk, such as individuals with a history of psychotic disorders or cardiovascular conditions. Preparatory sessions are used to set expectations for the effects of the drug. In the therapy sessions, a qualified guide can maintain a controlled environment, allowing the patient to safely reflect inward. Afterward, integration discussions are effective in helping patients talk through and process their experiences, increasing the probability of real, permanent change in behavior and mentality [12]. Further recommendations include standardized dosing protocols to reduce the risk of transient anxiety or physiological side effects [12]. Preparation and monitoring are essential in preventing psychological distress and maximizing psilocybin’s therapeutic potential.

## 5. Biochemistry

**Figure 4:**
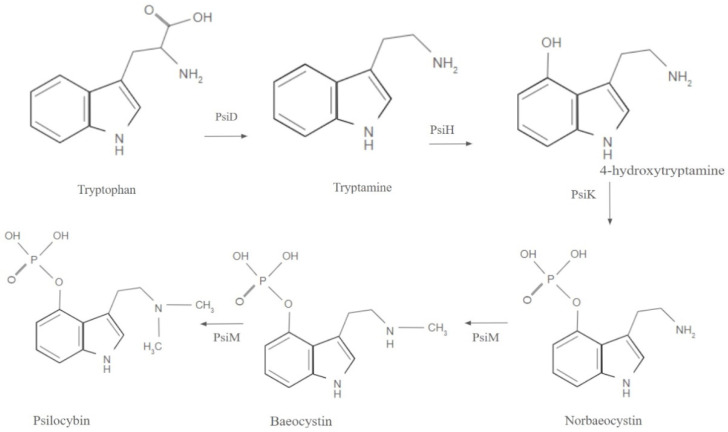
Biosynthetic pathway of psilocybin [21].

The biosynthetic pathway of psilocybin, which occurs in mushrooms of the Psilocybe genus, is depicted in Figure 4. Within the human body, it functions as a prodrug, which converts to psilocin through dephosphorylation by alkaline phosphatase in the stomach and intestines; psilocin is a more active metabolite capable of crossing the blood–brain barrier [5]. After ingestion, psilocybin is metabolized into psilocin, with 55% bioavailable and a half-life of 1.23–4.72 h [20].

Psilocin is subsequently metabolized in either of two phases. In Phase I, monoamine oxidase in the liver oxidizes psilocin to 4-hydroxyindole-3-acetaldehyde which is then either oxidized to 4-hydroxy-indole-3-acetic-acetic acid (HIAA) or reduced to 4-hydroxytryptophole [22,23]. In Phase II, psilocin is converted by the UDP-glucuronosyltransferases (UFT) 1A10 in the intestine and UFT1A9 in the liver to form O-glucuronide conjugates, which are then excreted in the liver [24,25,26].

Psilocin’s structural resemblance to serotonin enables it to activate 5HT-2A receptors, which triggers glutamate release in the prefrontal cortex and subsequent stimulation of metabotropic glutamate receptors 2 and 3 (mGluR2/3) [27].

By interacting with the serotonin and glutamate pathways, psilocybin has the potential to address cravings and cognitive dysfunction in addiction patients. Rats with decreased mGluR2 function experienced increased alcohol cravings [28]. Through modulation of the mGluR2 pathway, there is a potential treatment approach for alcohol addiction. Furthermore, by increasing extracellular glutamate, psilocybin increases the expression of brain-derived neurotrophic factor (BDNF), a protein associated with neuronal survival [27]. The BDNF and glutamate are thought to activate AMPA receptors, promoting neuroplasticity [27].

## 6. Pharmacokinetics

After ingestion of 15, 25, and 30 mg doses of psilocin, unconjugated psilocin reached peak plasma levels in 2 h, with elimination occurring through first-order kinetics [29]. The half-lives of the doses were 1.8, 1.4, and 1.8 h, respectively. Psilocin was metabolized into psilocin glucuronide and 4-HIAA with direct correlations between dose size and plasma concentrations. The first 8 h of urine analysis revealed that 75% of the total psilocin content and 61% of psilocin glucuronide as well as 81% of 4-HIAA content were recovered. About 1/3 of the ingested dose was eliminated as 4-HIAA and 20% as psilocin glucuronide. The amount of unconjugated, psychoactive psilocin recovered was 1.5% of the total dose, obtained in the first 24 h [29]. The renal clearance of psilocin averaged 42 ± 30 mL/min, and no significant correlation was found between plasma concentrations and factors such as body weight, BMI, GFR, or age [29].

## 7. Anti-Inflammatory Effects

Neuro-inflammation after TBI is a well-established process that is a potential target for intervention to improve patient outcomes. Psilocybin demonstrates a significant ability to reduce levels of the pro-inflammatory markers tumor necrosis factor-alpha (TNF-α) and interferon-gamma (IFN-γ) [30]. Additionally, psilocybin is capable of lowering interleukin-6 (IL-6) and interleukin-8 (IL-8), which stimulate acute-phase protein production and attract neutrophils [30]. The administration of psilocybin causes significant reduction in monocyte chemoattractant protein (MCP-1), which functions as an inflammatory site guidance mechanism for monocytes. Granulocyte–macrophage colony-stimulating factor (GM-CSF) serves as a protein that stimulates granulocyte and monocyte proliferation, but psilocybin reduces its expression [30]. A trial with 0.17 mg/kg of psilocybin measured glutamate and glial activity, finding an acute reduction in TNF alpha. Seven days later, TNF alpha had returned to baseline, but IL6 and CRP were still reduced. A correlation was found between the reduction in IL6 and CRP and positive mood and social effects reported by patients [31]. By acting on these various inflammatory mediators, psilocybin has potential as a broad-spectrum anti-inflammatory agent.

## 8. Plasticity

In the search for therapeutic interventions, neuroplasticity has emerged as a promising mechanism for recovery following TBI. It is theorized that the formation of new neurocircuitry could potentially compensate for damaged or lost neurons [32]. Medication-induced neuroplasticity offers the potential to repair damaged areas of the brain, which could lead to improved cognitive and motor functions [33,34]. After a single dose of psilocybin, mice had approximately 10% increases in dendritic spinal size and density [27]. This remodeling was found within 24 h of initial treatment and was still seen 1 month later [35]. These findings suggest psilocybin has clinical potential as a treatment that stimulates neuroplasticity.

Additionally, psilocybin has also demonstrated the ability to promote synaptogenesis in pig models. Psilocybin was administered at 0.08 mg/kg, and seven days later, hippocampal synaptic vesicle protein 2A (SV2A) density, an indicator of synapse quantity, was measured. An increase of 9.24% was found, indicating an increase in the number of synapses [36]. Raval’s study reveals psilocybin has the potential to increase neural connectivity, possibly even in TBI patients.

Another way psilocybin participates in neuronal remodeling is through high-affinity binding to tropomyosin receptor kinase B (TrkB). TrkB was found to promote neuroplasticity and was not inhibited by serotonin antagonists [28]. This pathway could eventually be targeted to promote psilocybin’s neuroprotective effects, independent of serotonin 2A receptor activation, removing the hallucinogenic side effects [37].

## 9. Cognitive Effects

Given the prevalence of cognitive impairments after TBI, psilocybin may have a promising therapeutic effect. In the acute phase, larger doses of psilocybin were found to impair cognitive performance but improve creativity [38,39]. However, these effects were found to be temporary, with no difference between psilocybin-treated and placebo-treated groups 85 days after treatment [40]. Through actions on the 5-HT2A receptor, psilocybin is thought to have an effect on memory, and imaging studies have been used to show that psilocybin use results in the activation of areas involved in memory [41,42].

However, there are conflicting results, with some studies showing no effect on memory, and other studies indicating a possible impairment of memory with medium and larger doses [43,44,45]. Most studies have investigated psilocybin intake in healthy participants over a relatively short period of time, with long-term effects poorly understood. To improve the understanding of long-term effects, there needs to be more research on the cognitive effects of psilocybin over greater lengths of time.

## 10. Psychological Effects

A significant number of TBI patients develop depression, which reaches 56.3% of survivors at 3 months after their injuries [46]. Research shows that major depressive disorder (MDD) occurs in 53% of patients during their first year following mild-to-severe TBI [30]. Available studies indicate that the general population has a depression rate of 6.7% [47].

The behavioral effects of psilocybin function similarly to standard psychedelic drugs because they activate the 5-hydroxytryptamine (5-HT)2A receptor. The receptor activity of psilocybin alone does not account for all its observed effects [48]. Studies propose that psilocybin works as a depression treatment by controlling brain activity. The medial prefrontal cortex (mPFC) becomes deactivated after psilocybin intake, according to functional MRI research, while this area typically displays excessive activity in people experiencing depression [42,49]. Other successful depression treatments demonstrate a similar ability to normalize hyperactivity in the mPFC, as observed in this study [49].

The administration of psilocybin leads to decreased amygdala activation when exposed to negative stimuli, thus minimizing adverse emotional reactions [50]. Research indicates that psilocybin diminishes the pathway between the amygdala and visual cortex, thus decreasing the brain’s reaction to visual danger [51]. The modification of emotion-processing pathways offers an alternate mechanism for psilocybin’s antidepressant properties.

Researchers have come to observe that psilocybin research has specific limitations. Carhart-Harris et al. [52] conducted an open-label trial in which participants were required to have had prior psychedelic experience. However, the unique psychotropic qualities of psilocybin make it difficult to create double-masked trials due to participant awareness of its effects. The previous experience of psilocybin required among study participants potentially increased their ability to detect its effects while simultaneously introducing pre-existing biases originating from their prior encounters. In order to develop a clear understanding of the therapeutic potential of psilocybin, these limitations must be addressed in future research.

A phase 2 trial proved that a single 25 mg dose of psilocybin in patients with treatment-resistant depression successfully lowered depression, as seen by a reduction of −12 in the MADRS score [53]. However, some participants also experienced adverse effects such as headache, nausea, and dizziness [53]. Due to concerns of ongoing SSRI use interfering with psilocybin treatment, another study followed 19 participants with ongoing SSRI treatment. After 3 weeks, they had an average Montgomery–Åsberg Depression Rating Scale change of −14.9 from baseline with no reports of serious or long-lasting treatment-emergent adverse events [15]. More significant, long-term studies are required to better understand the safety and efficacy of psilocybin compared with current standard medical approaches.

Bryant et al. [54] followed up with 1084 TBI patients at 3 months and 12 months after their injuries. The study showed PTSD incidence among patients reached 6% at 12 months, which was more likely if they had a mild TBI compared to no TBI (odds ratio 1.92, 85% CI = 1.08–3.40). The study by Anderson et al. [55] examined psilocybin-assisted group therapy for treating survivors of AIDS by showing positive results for both attachment anxiety and demoralization, symptoms of PTSD.

## 11. Adverse Effects

The metanalysis by Yerubandi et al. [56] established a link between psilocybin use and increased risk for various adverse effects, including headaches (RR 1.99, 95% CI 1.06–3.74, *p* = 0.04), nausea (RR 8.85, 95% CI 5.68–13.79, *p* < 0.001), anxiety (RR 2.27, 95% CI 1.11–4.64, *p* = 0.02), dizziness (RR 5.81, 95% CI 1.02–33.03, *p* = 0.047), and elevated blood pressure (RR 2.29, 95% CI 1.15–4.53, *p* = 0.02) [Table 1]. Research did not establish a connection between psilocybin use and the paranoia or transient thought disorder. All six reviewed studies documented headache and nausea as symptoms, but none of the other symptoms appeared in every study.

**Table 1:** Adverse effects found with psilocybin use and their mechanism of action.

Common Adverse Effects	Mechanism of Action
Headache	Possibly due to release of nitric oxide [57]
Nausea	Possibly the result of agonist action on 5-HT2A and 5-HT3 receptors affecting both central and peripheral serotonergic pathways [58,59]
Dizziness	Increases entropy of neural signaling, which can disrupt the ability to maintain a sense of balance and spatial orientation [60]
Elevated blood pressure	5-HT2A activation can result in vasoconstriction and increased cardiac output [58]

The research on both short-term and long-lasting adverse outcomes of psilocybin consumption remains insufficient. In a survey of 1993 individuals (mean age 30 years; 78% male) regarding their worst “bad trip” with psilocybin mushrooms, 39% identified these experiences as among the top 5 most difficult of their lives [57]; 11% of respondents admitted to endangering themselves or others [57], 2.6% exhibited aggressive behavior, and 2.7% ended up receiving medical help [57]. Among those whose psilocybin experiences occurred more than a year ago, 7.6% needed psychological treatment, including three cases of persistent psychotic symptoms and three of attempted suicide [57]. While challenging experiences correlated with higher doses, most respondents (84%) indicated they benefited from their experiences [45]. The data from controlled studies show that prepared participants experience limited distress during and after their participation [12].

## 12. Conclusions

The research on psilocybin as a therapeutic agent shows promise for its application in TBI in theory, but it requires more in-depth studies. Its anti-inflammatory properties and ability to promote neurogenesis and synaptogenesis suggests potential usefulness in TBI treatment. On top of that, the antidepressant properties can be used to address the high rates of depression in TBI patients. However, concerns regarding potential “bad trips” and other possible side effects stress the need for more controlled clinical trials to establish safe and effective protocols. Furthermore, it is important to consider if psilocybin will have a worsening effect on the sequelae seen in TBI patients. TBI is associated with an increased incidence of seizures [61], but a review of classic psychedelics in healthy human and animal models did not suggest an increased risk of seizures. This review did find an increased risk of seizure in those taking kambo or lithium, but authors of the review stated that their conclusions lacked external validity and recommended caution with interpretation [62]. No studies were found that explored the effects of psilocybin on autonomic dysfunction, a complication seen in TBI patients [63]. Further studies are also required to explore proper dosing within TBI patients, as TBI patients are at increased risk of BBB openings [64]. While decriminalization efforts in the United States are indicative of growing interest, its federal Schedule I classification limits rigorous scientific exploration. A combination of psilocybin treatment with current therapeutic practices has the potential to maximize TBI recovery, thus providing a novel method to enhance treatment for people dealing with this persistent condition.

## Data Availability

No new data were created or analyzed in this study. Data sharing is not applicable to this article.
